# Utilizing red blood cell distribution width (RDW) as a reliable biomarker to predict treatment effects after chimeric antigen receptor T cell therapy

**DOI:** 10.1007/s10238-024-01373-5

**Published:** 2024-05-21

**Authors:** Naokazu Nakamura, Tomoyasu Jo, Yasuyuki Arai, Toshio Kitawaki, Momoko Nishikori, Chisaki Mizumoto, Junya Kanda, Kouhei Yamashita, Miki Nagao, Akifumi Takaori-Kondo

**Affiliations:** 1 Department of Hematology, Kyoto University Hospital, Kyoto, Japan; 2 Department of Clinical Laboratory Medicine, Kyoto University Hospital, 54 Shogoin Kawahara-cho, Sakyo-ku, Kyoto, 606-8507 Japan; 3 Department of Human Health Sciences, Graduate School of Medicine, Kyoto University, Kyoto, Japan

**Keywords:** Chimeric antigen receptor T cell therapy, Biomarker, Red blood cell distribution width, Progression-free survival

## Abstract

Chimeric antigen receptor T cell (CAR-T) therapy is an effective treatment for B cell malignancies. A certain fraction of patients, however, experience post-CAR-T relapse, and due to the difficulty of precise relapse prediction, biomarkers that can predict the strength and duration of CAR-T efficacy are needed before CAR-T infusion. Therefore, we performed a single-center cohort study including 91 diffuse large B cell lymphoma (DLBCL) patients treated with CAR-T in order to identify such a new prognostic biomarker. After confirming that each of the already reported prognostic parameters (disease status at leukapheresis, primary refractoriness, number of treatment lines, CD3^+^ cell counts at leukapheresis) has only limited predictive performance, we established a new composite parameter by integrating these four variables, and found that it predicts progression-free survival (PFS) after CAR-T infusion with statistical significance. Moreover, after comprehensive correlation analyses of this new composite parameter with all individual laboratory variables, we determined that the standard deviation of red blood cell distribution width (RDW-SD) at leukapheresis shows significant correlation with the composite parameter and may be a prognostic biomarker (*R*^2^ = 0.76, *p* = 0.02). Validation analysis indicated that a higher RDW-SD is significantly associated with poorer PFS after CAR-T cell therapy (HR, 3.46, *P* = 0.03). Thus, this study suggests that a single parameter, RDW-SD at leukapheresis, is a novel, useful biomarker that can be obtained early to predict therapeutic effects of CAR-T cell therapy. Post-CAR-T maintenance or re-induction therapies should be adopted for higher risk patients, who may relapse after CAR-T therapy.

**Supplementary Information:**

The online version contains supplementary material available at 10.1007/s10238-024-01373-5.

## Introduction

Chimeric antigen receptor T cell (CAR-T) therapy has become one of the most effective treatments for relapsed or refractory (r/r) B cell malignancies, including B cell acute lymphoblastic leukemia (B-ALL), B cell lymphomas, and multiple myeloma (MM) [1–4]. However, not all patients enjoy sufficient and prolonged efficacy of CAR-T cell therapy. Patients who fail to respond to CAR-T cell therapy or who experience post-CAR-T relapse have dismal prognoses [5]. Hence, optimization of comprehensive treatment strategies for r/r B cell malignancies must cover post-CAR-T relapse. Such strategies may comprise multimodal treatment, including cytotoxic chemotherapy, cytotherapy, bispecific T cell engager (BiTE), and other immune regulatory agents. Therefore, prediction of CAR-T effects, both short- and long-term, is extremely important.

In previous studies, several factors have been advocated in relation to outcomes after CAR-T cell therapy, such as disease status, residual disease volume, and host factors, as well as potency of CAR-T cells [6, 7]. However, none of these previously reported factors individually is sufficiently robust, and they must be combined in a complicated manner to more precisely predict prognosis after CAR-T cell therapy. Moreover, most of these parameters can only be obtained right before CAR-T infusion, and long-term therapeutic strategies cannot be determined in advance, including the most appropriate timing for CAR-T infusion or the conduct itself of CAR-T cell therapy. In clinical practice, more accurate, convenient, and earlier prediction of CAR-T-effects will enable physicians to provide patients with more appropriate treatment before CAR-T therapy and risk-adapted follow-up after CAR-T therapy [8–10].

In this study, we sought to identify a reliable biomarker that can predict therapeutic efficacy of CAR-T cell therapy as early as leukapheresis. We focused on laboratory data at leukapheresis in relation to prognostic outcomes after CAR-T cell therapy, and identified red blood cell distribution width (RDW) as a novel, predictive biomarker. Its clinical use will improve cytotherapy management and outcomes of CAR-T cell therapy.

## Methods

### Patients

This retrospective study enrolled all consecutive adult (≥ 16 years) patients with relapsed or refractory diffuse large B cell lymphoma (DLBCL) who received CD19-targeting CAR-T therapy (tisagenlecleucel [tisa-cel], lisocabtagene maraleucel [liso-cel], or axicabtagene ciloleucel [axi-cel]) from February 2019 to June 2023 at Kyoto University Hospital. Patients for whom response data were unavailable were excluded. This study was approved by the Institutional Review Board and Ethic Committee of Kyoto University. Written informed consent was obtained from all participating patients.

## Endpoints and definitions

The primary endpoint of this study was progression-free survival (PFS). The secondary endpoint was overall survival (OS). PFS was defined as the time from CAR-T infusion to relapse, progression, death, or the last follow-up date. OS was defined as the time from CAR-T infusion to death or the last follow-up date. The RDW standard deviation (RDW-SD) and RDW coefficient of variation (RDW-CV) in peripheral blood were measured using a Sysmex XN-10-B3 (Sysmex, Japan). Diagnosis of DLBCL was based on the WHO classification of tumors of hematopoietic and lymphoid tissues (revised 4th edition) [11]. Cell-of-origin classification was determined by immunostaining of CD10, BCL6, and MUM1 based on the Hans classifier [12], and CD5 expression was evaluated by immunostaining. Disease status was evaluated at leukapheresis with FDG-PET/CT, using the revised response criteria for malignant lymphoma [13–15]. Before CAR-T cell infusion, patients received lymphodepletion (LD) chemotherapy, including fludarabine with cyclophosphamide, and bendamustine-based regimens, according to the manufacturers’ instructions [16–18]. Diagnosis and grading of cytokine-release syndrome (CRS) and immune-effector cell-associated neurotoxicity syndrome (ICANS) followed guidelines of the American Society for Transplantation and Cellular Therapy [4, 19]. Vascular risks relevant to RDW included history of ischemic heart disease, cerebral infarction, arteriosclerosis obliterans, deep vein thrombosis, atrial fibrillation, diabetes mellitus, chronic kidney disease, and other cancers [20–23].

### Statistical analysis

Continuous variables were summarized using medians and ranges, and categorical variables were summarized as counts and percentages. For comparisons between groups, patient and disease characteristics were compared using Student’s t test or ANOVA for continuous variables, and Fisher’s exact test for categorical variables. Probabilities of PFS and OS were estimated using the Kaplan–Meier method and compared between groups with the Cox proportional-hazards model. Comprehensive correlation between individual laboratory markers (list in Supplemental Table S1) and the composite parameter was evaluated using the Pearson correlation coefficient. Multiple regression analysis was used to assess relationships between RDW-SD and six variables available at leukapheresis [disease status (complete remission (CR) or partial remission (PR) vs. stable disease (SD) or progression disease (PD)], whether the case was primary refractory, coexistent vascular risk, number of treatment lines, hemoglobin (Hb) levels, and CD3^+^ cell counts). Accuracy of prediction with an approximate formula obtained from multiple regression analysis was assessed with Fisher’s exact test. Statistical significance was set at *p* < 0.05. All statistical analyses were performed using EZR software (Saitama Medical Center, Jichi Medical University, Saitama, Japan) [24].

## Results

### Patient characteristics

Table 1 presents characteristics of patients. We enrolled 91 patients treated with tisa-cel (*N* = 70), liso-cel (*N* = 15), or axi-cel (*N* = 6) for r/r DLBCL. Median age at leukapheresis was 62 years (range, 19–75). Eastern cooperative oncology group performance status (ECOG-PS) at leukapheresis was 0 in 85 (93.4%), 1 in 5 (5.5%), and 2 in 1 (1.1%). No patient had active infections at leukapheresis. Median number of treatment lines before CAR-T cell infusion was 4 (2–7). Bispecific antibody was not used in our cohort, and polatuzumab vedotine was included as a prior treatment in 90 (98.9%) cases. Disease status at leukapheresis and before CAR-T cell infusion was SD or PD in 19 (20.9%) and 18 (19.8%), respectively. After CAR-T cell infusion, CRS was observed in most patients (*N* = 83, 91.2%). ICANS was observed in 14 (15.4%). In total, tocilizumab was administered to 74 patients (81.3%) and 25 (27.5%) received corticosteroids for CRS or ICANS. Median follow-up time after infusion was 378 days.

**Table 1:** Patient characteristics

Variables	*N* = 91 (%)
Median age (range)	62 (19–75)
Sex (Female/Male)	50 (54.9%)/41 (45.1%)
Details	
IPI (Low/intermediate/high)	3 (3.3%)/36 (39.6%)/52 (57.1%)
CD5 expression	18 (19.8%)
Double expresser of MYC/BCL2	29 (31.9%)
Double hit of MYC/BCL2	10 (21.3%)
ECOG-PS (0/1/2-)	85 (93.4%)/5 (5.5%)/1 (1.1%)
CAR-T product	
Tisa-cel/liso-cel/axi-cel	70 (76.9%)/15 (16.5%)/6 (6.6%)
Treatment lines before CAR-T, median (range)	4 (2–7)
Disease status at leukapheresis	
CR/PR/SD/PD	4 (4.4%)/68 (74.7%)/2 (2.2%)/17 (18.7%)
Disease status right before LD chemo	
CR/PR/SD/PD	24 (26.4%)/49 (53.8%)/1 (1.1%)/17 (18.7%)
CRS (grade 1/2/3–4)	71 (78.0%)/7 (7.7%)/5 (5.5%)
ICANS (grade 1/2-)	3 (3.3%)/11 (12.1%)
CRS/ICANS treatment	
Tocilizumab/corticosteroid	74 (81.3%)/25 (27.5%)

*IPI* international prognostic index, *ECOG-PS* Eastern Cooperative Oncology Group Performance Status, *CAR-T* chimeric antigen receptor T cell, *Tisa-cel* tisagenlecleucel, *Liso-cel* lisocabtagene maraleucel, *Axi-cel* axicabtagene ciloleucel, *CR* complete remission, *PR* partial remission, *SD* stable disease, *PD* progression disease, *LD chemo* lymphocyte depletion chemotherapy, *CRS* cytokine-release syndrome, *ICANS* immune-effector cell-associated neurotoxicity syndrome

### Association of previously reported biomarkers and survival after *CAR*-T cell therapy

**Fig. 1:**
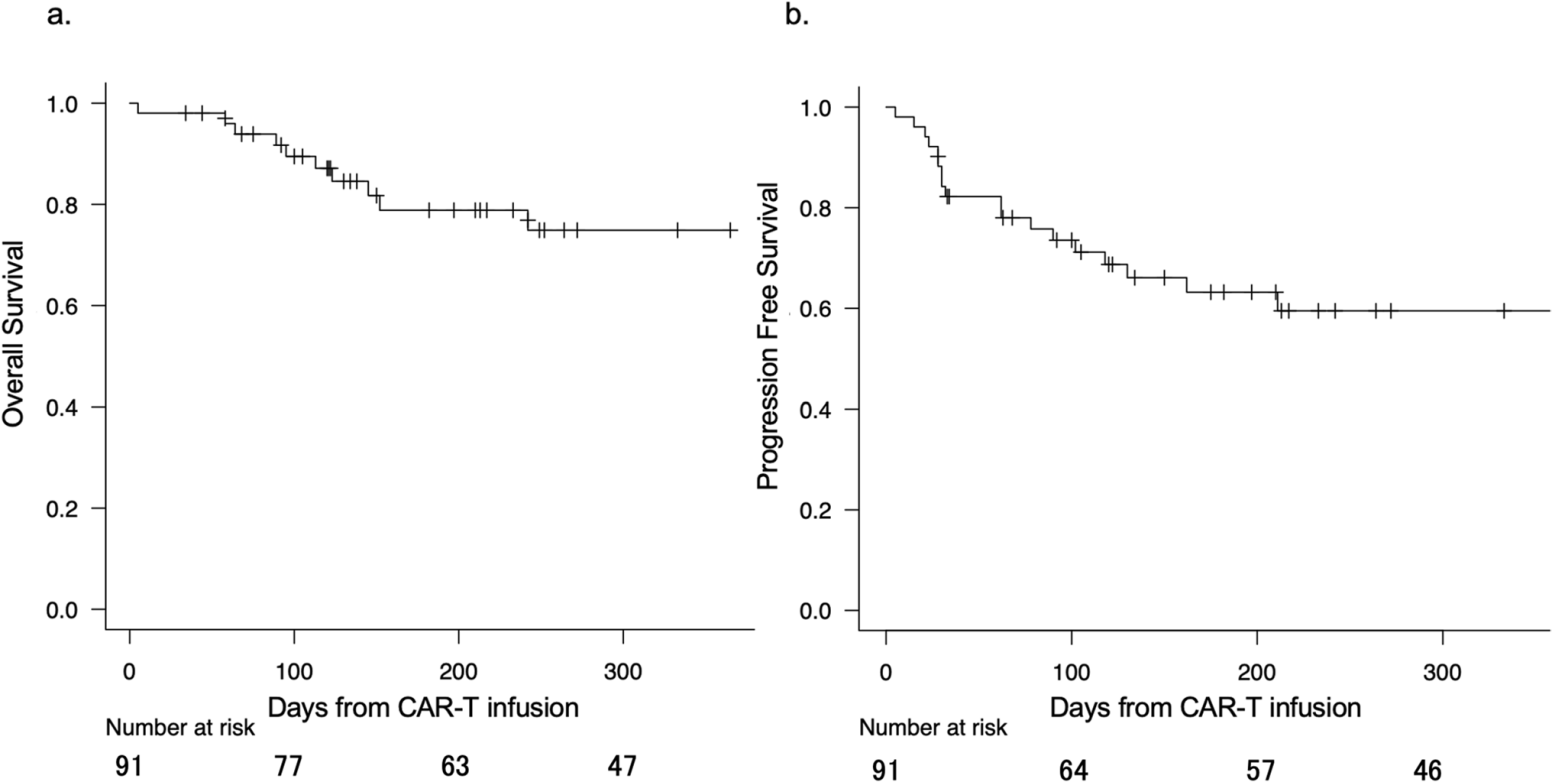
Entire prognosis after CAR-T infusion. Kaplan–Meier estimates of overall survival (**a**) and progression-free survival (**b**) among the entire cohort

Among all patients, 1-year OS and PFS rates were 77.7% and 60.3% (Fig. 1a, b). First, we checked the predictive performance of extant biomarkers in our cohort. In patients whose disease status was SD or PD at leukapheresis, the 1-year PFS rate tended to be worse (43.2%) than in patients whose disease status was CR or PR (76.3%, hazard ratio [HR], 1.91, 95% confidence interval (CI), 0.92–3.47, *P* = 0.08; Figure S1a). In patients with a history of primary refractory diseases, 1-year PFS tended to be worse (40.0%) than in patients without (77.5%, HR, 1.86, 95% CI, 0.93–3.33, *P* = 0.09; Figure S1b). Higher numbers of treatment lines were marginally associated with worse PFS (HR, 1.19 per each line, 95% CI, 0.90–1.45 per each line, *P* = 0.09; Figure S1c). As has been reported previously in B cell lymphomas [7], a lower CD3^+^ cell count at leukapheresis was associated with worse PFS (HR, 0.75 per 100 cells/μL, 95% CI, 0.50–1.04, *P* = 0.06; Figure S1c). These data suggest that previously reported biomarkers are individually associated with PFS; however, each biomarker can be confounded by others. For example, disease status at leukapheresis was significantly associated with CD3^+^ cell counts (median, 724/μL in CR/PR; 432/μL, *P* = 0.03), but it is also associated with a history of primary refractory disease (7.2% in CR/PR; 17.8% in SD/PD, *P* = 0.03). Appropriate adjustments for confounding are required to evaluate true impacts on prognosis.

Therefore, we performed logistic analyses for PFS at 1 year after CAR-T infusion using these 4 parameters and found them significant or marginally significant (Table 2). According to these results, probability of PFS at 1 year (indicated as *p*) was mathematically estimated as follows: log(*p*/1 − *p*) = 0.29 × (1 if disease status at leukapheresis was SD or PD; 0 if it was CR or PR) + 0.31 × (1 if with a history of primary refractory disease; 0 without) + 0.11 × (number of treatment lines) − 0.0031 × (CD3^+^ cell count in peripheral blood [/μL]) − 0.28. Although this newly established composite parameter (right side of the above equation) can eliminate confounding between variables and can predict PFS after CAR-T treatment more accurately than individual parameters, its complex calculation is not acceptable in a clinical setting, and some simpler surrogate biomarker needs to be identified.

**Table 2:** Multivariate logistic analyses regarding PFS at 1 year after CAR-T

Variables	OR (95% CI)	*P* value
Disease status at leukapheresis		
SD, PD vs CR, PR	1.30 (1.08–2.67)	0.03
A history of primary refractory disease		
Yes vs No	1.37 (1.11–3.33)	0.02
Number of treatment lines		
Per one line	1.12 (0.98–2.22)	0.06
CD3^+^ cell count in peripheral blood		
Per one cell/uL	0.997 (0.993–1.001)	0.07

*OR* odds ratio, *95% CI* 95% confidential interval, *SD* stable disease, *PD* progressive disease, *CR* complete remission, *PR* partial remission

### Relationship between multiple prognostic factors and RDW-SD at leukapheresis

Next, we tried to identify a single surrogate biomarker to replace the aforementioned composite parameter, and performed comprehensive correlation analyses. Significant correlations with RDW-SD and RDW-CV were observed (RDW-SD; contribution rate (*R*^2^) = 0.67, *P* = 0.04, RDW-CV; *R*^2^ = 0.63, *P* = 0.05, Figure S2). Since RDW-SD and RDW-CV were strongly correlated [Pearson correlation coefficient (PCC), 0.878, *P* < 0.01, Figure S3], as expected, we selected RDW-SD as representative of RDW in subsequent analyses. RDW-SD values at first diagnosis and leukapheresis were not correlated (PCC, 0.379, *P* = 0.29).

On the basis of the correlation analysis, RDW-SD at leukapheresis was explained by the four prognostic parameters as well as additional two additional parameters, hemoglobin (Hb) levels and vascular risks, which were reported to affect RDW-SD from the viewpoint of erythrocyte biology: Estimated value of RDW-SD (fL) = 7.39 × (1 if disease status at leukapheresis in SD or PD; 0 if in CR or PR) + 14.25 × (1 if with a history of primary refractory disease; 0 if without) + 3.52 × (1 for vascular risk; 0 for others) + 4.10 × (number of treatment lines) − 0.75 × (Hb level [g/dL]) − 0.0042 × (CD3^+^ cell count in peripheral blood [/μL]) + 38.24. These estimated values were significantly correlated with the actual values (*R*^2^ = 0.76, *P* = 0.02, Figure S4). With this approximate formula, it is easier to distinguish the expected high-value group from the expected low-value group, and high RDW-SD (≥ 51 fL) and low RDW-SD (< 51 fL) were predicted with accuracies of 82.1 and 90.4%, respectively (*P* < 0.01, Table S2). These findings suggest that RDW-SD at leukapheresis is a biomarker strongly influenced by various clinical factors.

### Higher RDW-SD at leukapheresis predicts poorer PFS after *CAR*-T cell therapy

**Fig. 2:**
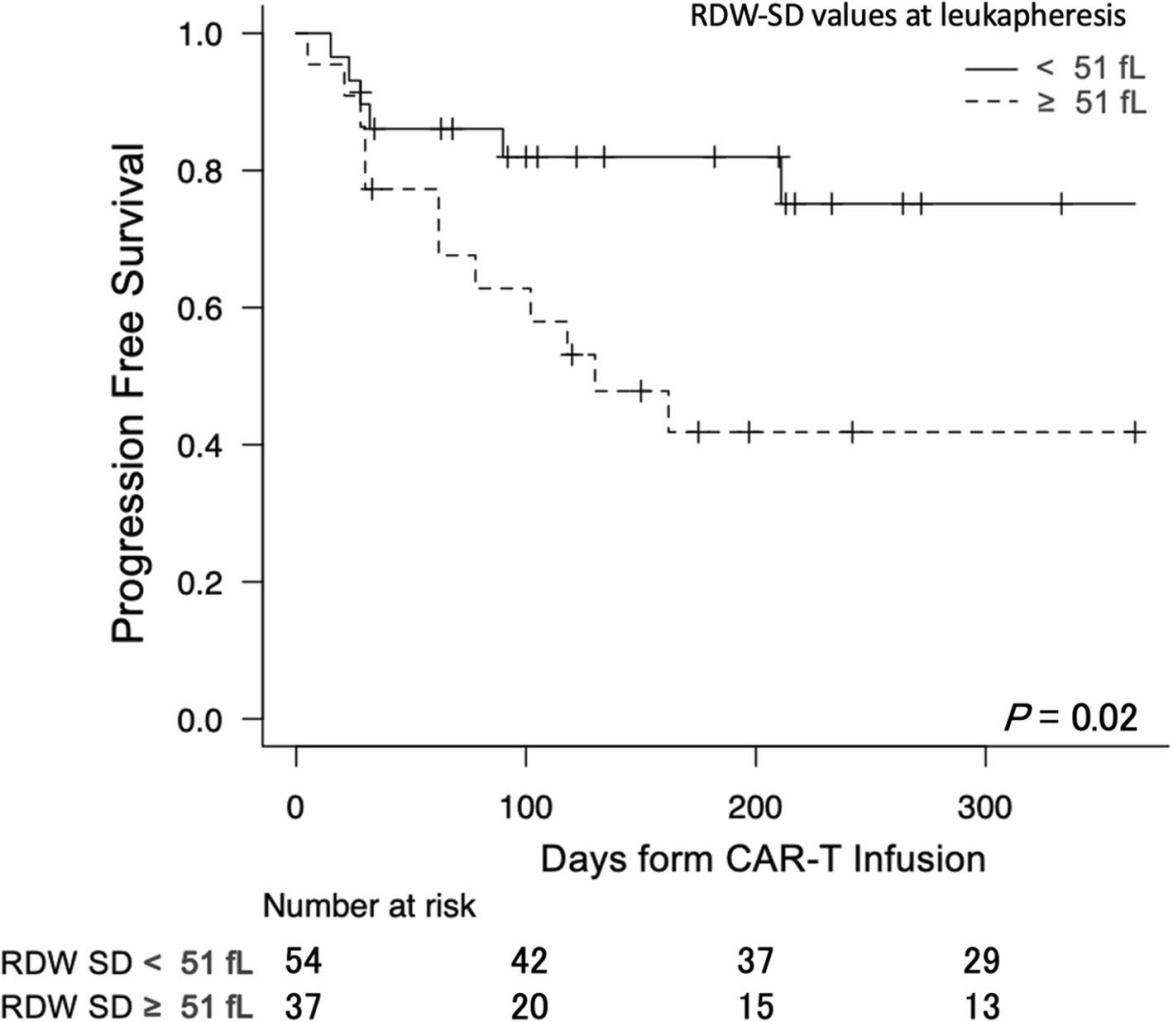
Kaplan–Meier estimate of PFS according to RDW-SD values at leukapheresis. The whole patient cohort was divided into two groups according to RDW-SD values at apheresis (higher vs lower than the threshold of 51 fL), and Kaplan–Meier curves were calculated in each subgroup

Since we had found a strong correlation between RDW-SD and other parameters (conventionally known prognostic parameters, as well as other patient parameters), we then assessed RDW-SD at leukapheresis as a predictor of survival after CAR-T cell therapy. PFS diminished significantly with higher RDW-SD, when treating it as a continuous variable (HR, 1.78 per each fL, 95% CI, 1.11–2.56, *P* = 0.02). Moreover, when patients were divided into two groups according to RDW-SD at leukapheresis, patients with high RDW-SD (≥ upper limit of normal range [51 fL]) showed worse PFS than those with low RDW-SD (< 51 fL) (HR 2.39; 95% CI, 1.44–5.21; *P* = 0.02, Fig. 2).

## Discussion

A reliable biomarker to predict treatment responses to CAR-T therapy, well before initiation of such therapy, is urgently required to optimize treatment against r/r DLBCL. In this retrospective cohort study, we found the following: (1) RDW at leukapheresis integrates existing biomarkers for PFS after CAR-T cell therapy, and (2) RDW at leukapheresis more easily and reliably predicts treatment outcomes of DLBCL patients after CAR-T therapy, than existing biomarkers.

First, we evaluated utility of previously reported factors (poor disease status, history of primary refractory disease, a higher number of treatment lines, CD3^+^ cell counts at leukapheresis) to predict PFS, and found that individually, these factors were inadequate due to their poor predictive power and strong confounding. Although a composite parameter combining these factors in multivariate analyses can overcome these problems, the necessary formula is too complicated to be applied in clinical practice. Therefore, we searched for a single, surrogate marker to replace the aforementioned four prognostic markers using patient variables at leukapheresis. We identified high RDW-SD as a promising biomarker that is strongly correlated with the composite. Moreover, RDW-SD at leukapheresis was accurately estimated using the 4 extant prognostic markers and erythrocyte-related factors, suggesting that RDW-SD integrates existing prognostic markers. Then, we showed that higher RDW-SD at leukapheresis predicts poorer PFS after CAR-T cell therapy.

RDW is a readily measurable laboratory parameter of heterogeneity in red blood cell size [25–27]. Both RDW-SD and RDW-CV quantify variation of red blood cell size. RDW-SD is calculated from the diameter of the erythrocyte distribution curve at 20% above baseline, and is expressed per femtoliter (fL) [28–30]. The coefficient of variation of erythrocyte volume histogram is expressed in % [31–33]. Originally, RDW was used to evaluate anisocytosis of red blood cells and to differentiate causes of anemia [34–38]. Recently, however, RDW has been employed in various clinical fields. For instance, in cardiovascular disease, vascular endothelial disorder causes elevated RDW [20–23]. Moreover, it has been reported that elevated RDW level is associated not only with vascular risks, but also prognosis of various cancers, such as breast cancer, lung cancer, and esophageal cancer [39–44]. However, the connection between RDW in hematological malignancy and cellular therapy has been unclear [45]. Here, we showed that higher RDW is significantly associated with poorer PFS after CAR-T cell therapy in patients with B cell malignancies.

While mechanisms underlying the relationship between high RDW and poor prognosis in patients after CAR-T cell therapy remain unclear, a potential explanation is as follows. Several researchers hypothesized that chronic inflammation and reactive oxygen species (ROS) stress may elevate RDW [46–48]. Chronic inflammation and ROS stress can lead to increased RDW by suppressing effective bone marrow erythropoiesis, increasing red blood cell variability [49–51]. Oxidative stress decreases erythrocyte survival and leads to increased numbers of circulating premature erythrocytes, resulting in anisocytosis and higher RDW [52–54]. Therefore, high RDW may reflect chronic inflammation due to repeated chemotherapy, infection, and progressive underlying disease. In fact, our study revealed that patients with high RDW levels had undergone more treatment lines for chemo-resistant disease, and had anemia and decreased CD3^+^ cell counts. Moreover, we demonstrated that RDW-SD was well estimated by 6 clinical parameters. Therefore, RDW-SD, determined by a simple laboratory test, integrates clinical factors and can be used as a biomarker for efficacy of CAR-T cell therapy.

There are some limitations in this study. First, this is a single-center study, involving small number of patients with heterogeneous backgrounds. Due to its retrospective nature, potential confounding factors may not be well enough measured or adjusted. Future validation is warranted. Second, the impact of anemia on RDW-SD has not completely been eliminated. The median hemoglobin level at leukapheresis was 10.9 g/dL in this study, and anemia is induced by several factors, including prior treatments and chronic inflammation. Of 91 patients, 13 (14.3%) received red blood cell transfusions a day before leukapheresis to increase collection efficiency of leukapheresis. Therefore, we selected the values right before leukapheresis to minimize effects of prior treatments and we used the values right before transfusions to eliminate the influence of transfusions in the 13 cases. Third, RDW-SD is influenced by other factors. No patients were checked for vitamin B12, folic acid, iron, or ferritin at leukapheresis. Other comorbidities can impact on RDW-SD such as heart diseases, lung diseases, diabetes, hypertension, and chronic inflammatory diseases. We included patients with these comorbidities, but these were not related to RDW-SD values nor PFS.

In summary, our study revealed that RDW at leukapheresis integrates clinical variables at leukapheresis, and can be used as a convenient, useful biomarker to predict outcome of CAR-T therapy early in treatment of DLBCL. Our results not only shed light on clinical backgrounds underlying RDW’s utility in prognosis for patients with hematological malignancies, but also helps to optimize CAR-T cell therapy for these patients.

## Supplementary Information

Below is the link to the electronic supplementary material.

Supplementary file1 (PDF 1491 KB)

## Data Availability

Data that support the findings of this study are available from the corresponding author upon request.
