# Supplementary material for: Utilizing red blood cell distribution width (RDW) as a reliable biomarker to predict treatment effects after chimeric antigen receptor T cell therapy
Source: Clin Exp Med. 2024 May 21;24(1):105. doi: 10.1007/s10238-024-01373-5 (PMC11108946; doi:10.1007/s10238-024-01373-5)
Supplement: Supplementary file 1 — Supplementary file1 (PDF 1491 KB) [file 10238_2024_1373_MOESM1_ESM.pdf]

Supplemental Materials

Table S1. List of laboratory markers in comprehensive correlation analyses

1.	WBC (/mm^3)	11.	FRC (%)
2.	Neut (/mm^3)	12.	Plt (×10^3/μL)
3.	Lymph (/mm^3)	13.	IPF (%)
4.	Hb (g/dL)	14.	PT-INR
5.	MCV (fL)	15.	APTT (s)
6.	MCHC (%)	16.	D-dimer (μg/mL)
7.	Reti (‰)	17.	LDH (U/L)
8.	RET-He (pg)	18.	Cre (mg/dL)
9.	RDW-SD (%)	19.	CRP (mg/dL)
10.	RDW-CV (fL)	20.	Ferritin (ng/mL)

Abbreviations: WBC, white blood cell count; Neut, neutrophil count; Lymph, lymphocyte count; Hb, hemoglobin; MCV, mean corpuscular volume; MCHC, mean corpuscular hemoglobin concentration; Reti, reticulocyte; Ret-Hb; reticulocyte hemoglobin equivalent; RDW-SD, red blood cell distribution width-standard deviation; RDW-CV, red blood cell distribution width-coefficient of variation; FRC, fragmented red blood cell; Plt, platelet count; IPF, immature platelet fraction; PT-INR, prothrombin time-international normalized ratio; APTT, activated partial thromboplastin time; D-dimer, LDH, lactate dehydrogenase; Cre, creatinine; and CRP, C-reactive protein.

Table S2. Validation of the estimated value of RDW-SD

		Estimated value		
		< 51 fL	≥ 51 fL	Total
Actual value	< 51 fL	47 (90.4%)	7 (17.9%)	54 (59.3%)
	≥ 51 fL	5 (9.6%)	32 (82.1%)	37 (40.7%)
	Total	52 (100%)	39 (100%)	91 (100%)

P < 0.001 (Fisher's test)

Supplementary Figure Legends

Figure S1. PFS according to the already known biomarker

PFS differences according to disease status (a) and primary response (b) are shown. Correlations of PFS with number of treatment line and CD3+ counts are shown in the tabular form (c).

Figure S2. The correlation of the composite parameter (integrating four prognostic factors) and several laboratory parameters at leukapheresis

Abbreviations are shown in Table S1.

Figure S3. Correlation between RDW-SD and RDW-CV

Correlation with RDW-SD and -CV is shown. Each dot indicates each patient data.

Figure S4. Correlation between actual value and estimated value of RDW-SD

RDW-SD values actually measured before apheresis were plotted in the X axis, while those estimated by 6 relevant parameters are shown in the Y axis. Each dot indicates each patient.

Figure S1

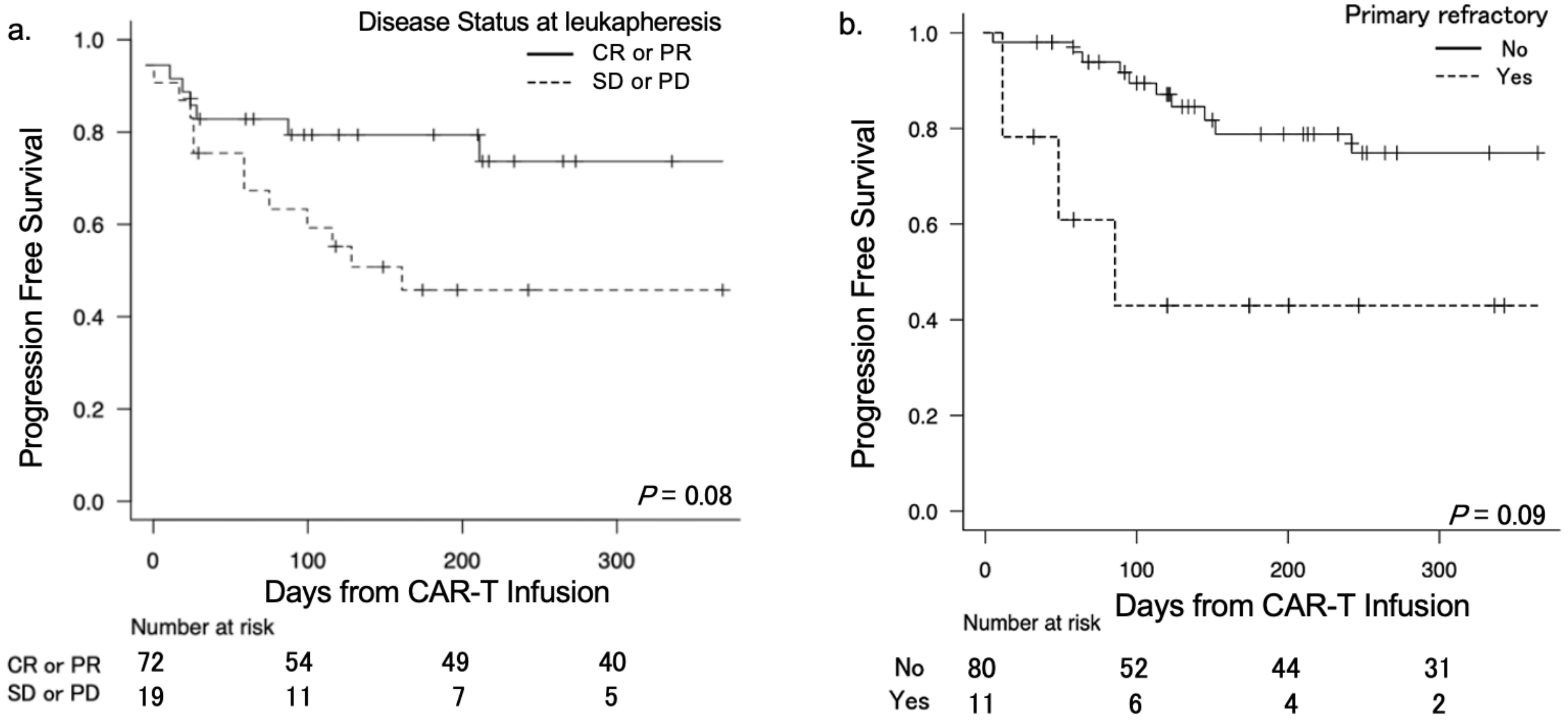

c.

	Progression or Relapse or Death	
**Parameters before lymphocyte apheresis**	**HR (95% CI)**	**P**
Number of treatment lines	1.19 (0.90-1.45)	0.09
$CD3^+$ cell count (per 100/μL) in peripheral blood	0.75 (0.50-1.04)	0.06

Figure S2

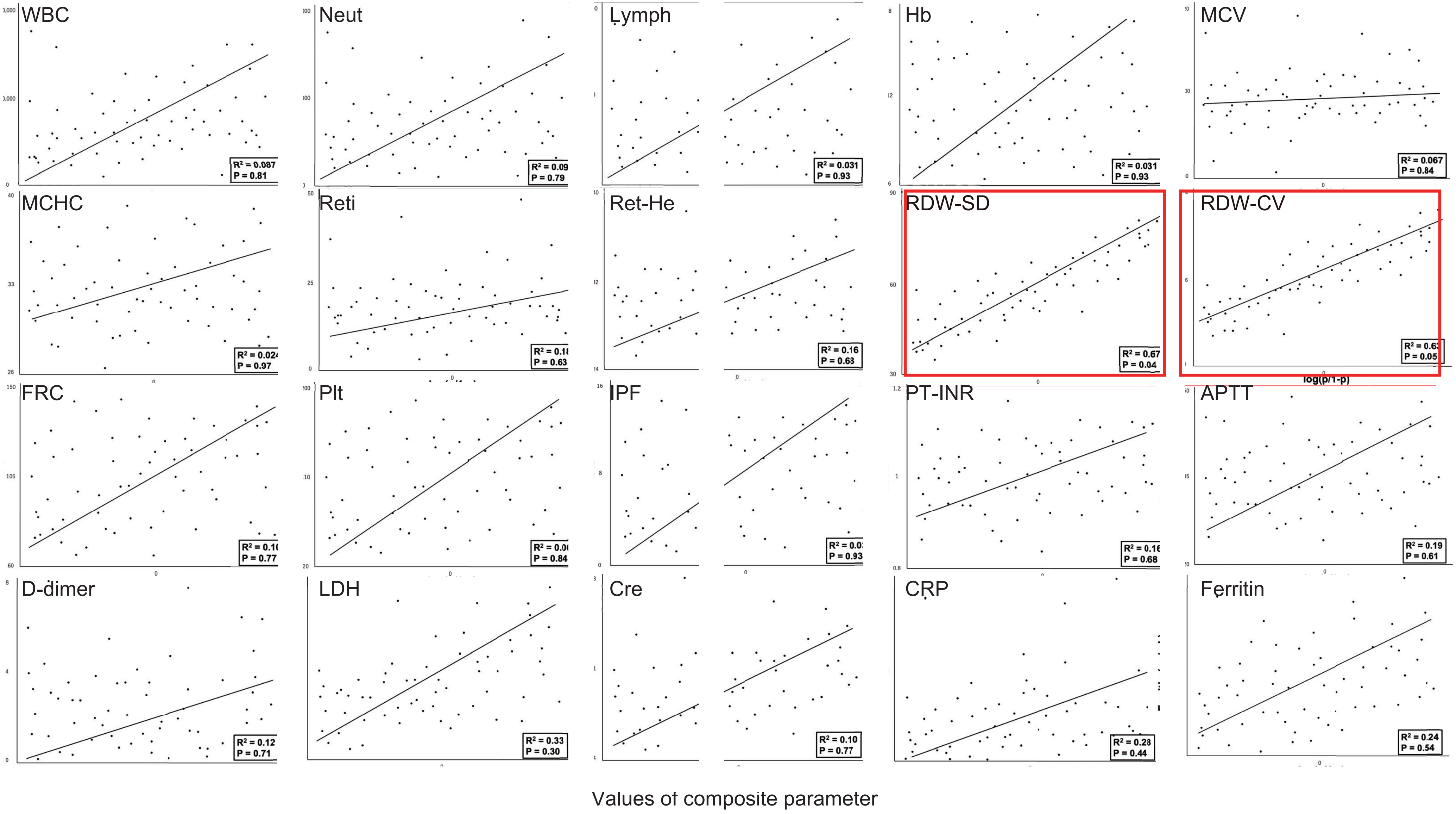

WBC
R² = 0.087
P = 0.81
Neut
R² = 0.09
P = 0.79
Lymph
R² = 0.031
P = 0.93
Hb
R² = 0.031
P = 0.93
MCV
R² = 0.067
P = 0.84
MCHC
P = 0.97
Reti
P = 0.63
Ret-He
R² = 0.16
P = 0.68
RDW-SD
R² = 0.67
P = 0.04
RDW-CV
P = 0.05
log(p/1-p)
FRC
P = 0.77
Plt
P = 0.84
IPF
P = 0.93
PT-INR
R² = 0.16
P = 0.68
APTT
R² = 0.19
P = 0.61
D-dimer
R² = 0.12
P = 0.71
LDH
R² = 0.33
P = 0.30
Cre
R² = 0.10
P = 0.77
CRP
R² = 0.28
P = 0.44
Ferritin
R² = 0.24
P = 0.54
Values of composite parameter

Figure S3

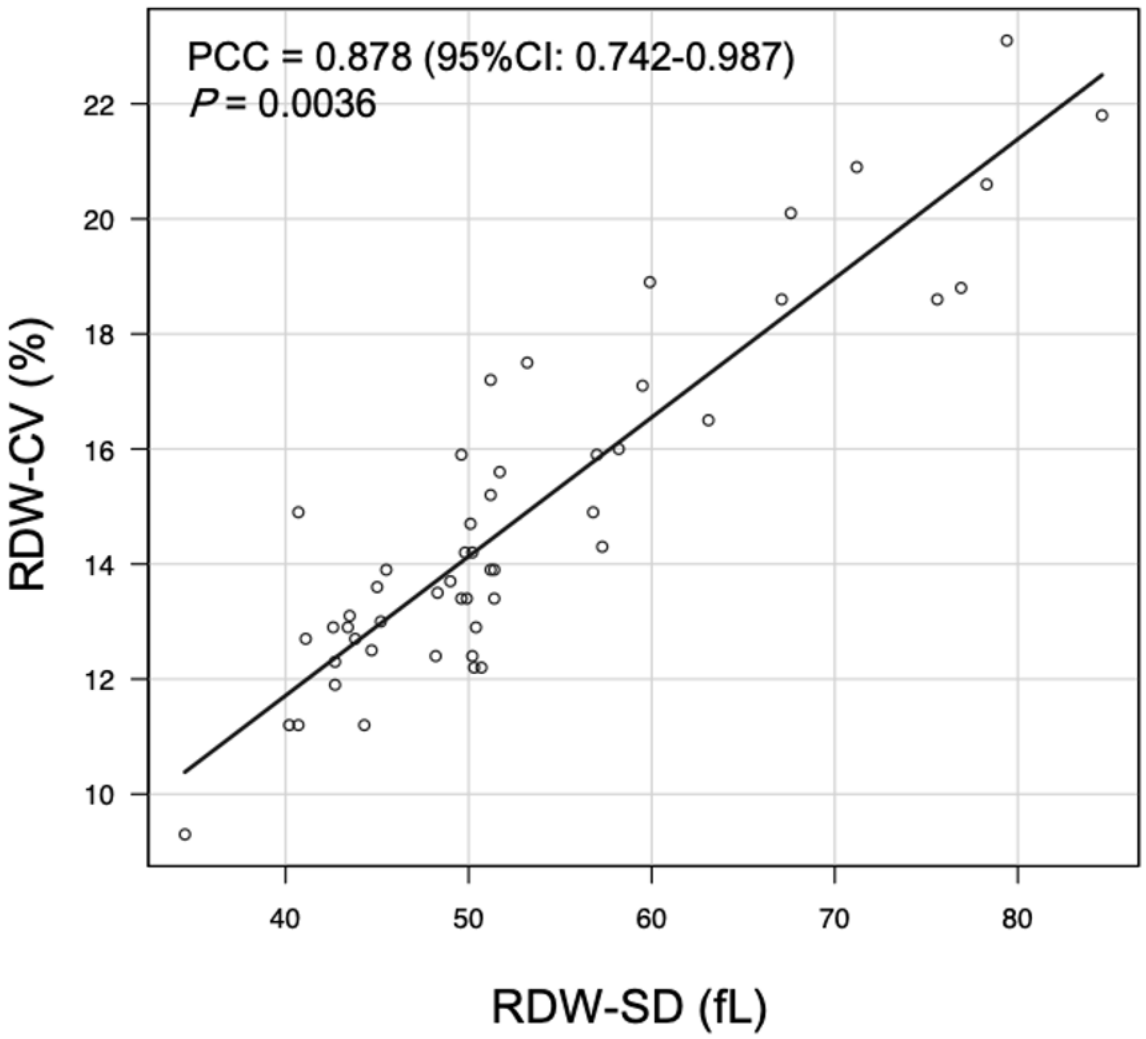
PCC = 0.878 (95%CI: 0.742-0.987)
P = 0.0036
RDW-CV (%)
RDW-SD (fL)
22
20
18
16
14
12
10
40
50
60
70
80

Figure S4

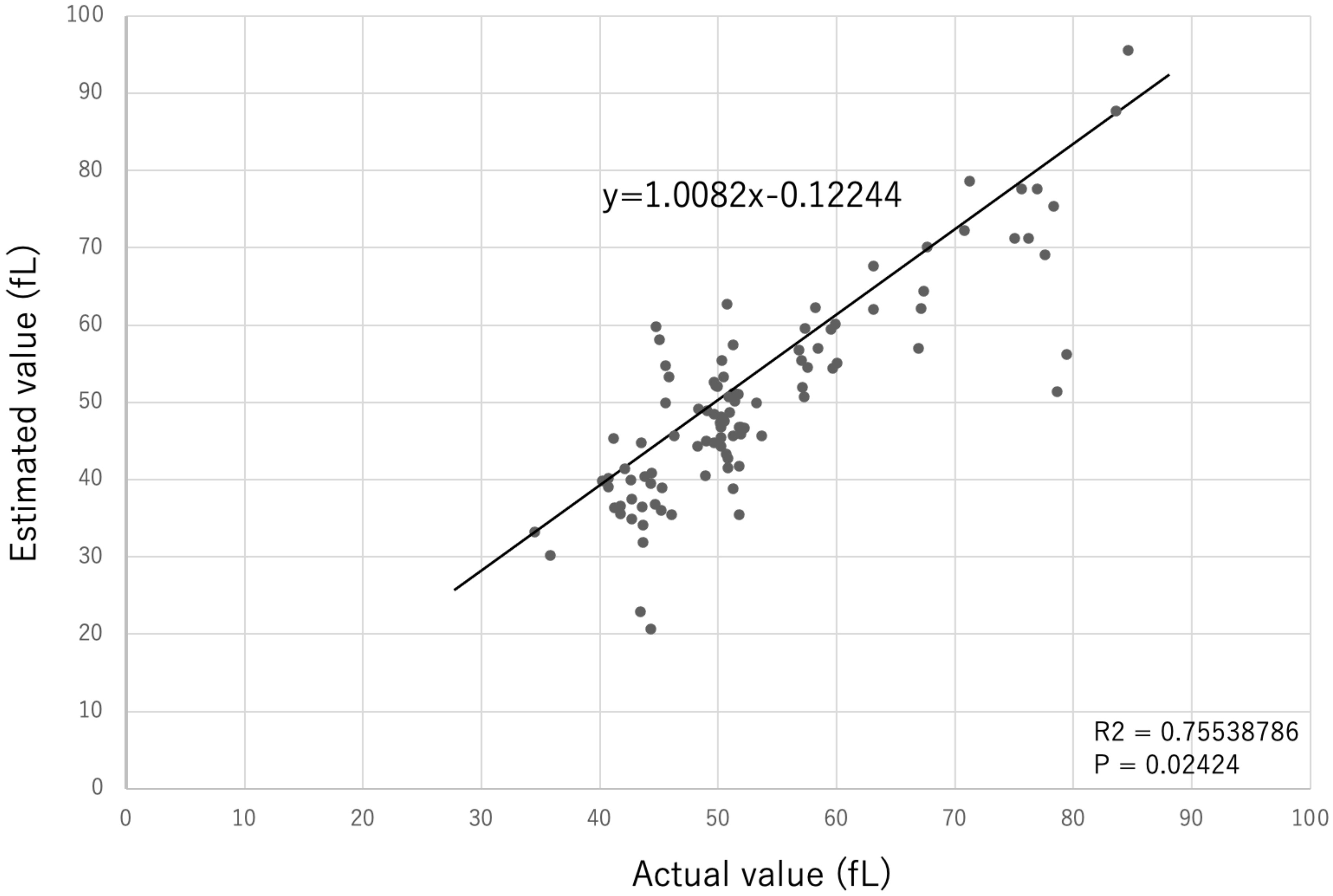
y=1.0082x-0.12244
R2 = 0.75538786
P = 0.02424
Estimated value (fL)
Actual value (fL)
0
10
20
30
40
50
60
70
80
90
100
